# Shift in epitope dominance of IgM and IgG responses to *Plasmodium falciparum *MSP1 block 4

**DOI:** 10.1186/1475-2875-9-14

**Published:** 2010-01-13

**Authors:** Sandra P Chang, Alexander KK Kayatani, Zilka I Terrientes, Socrates Herrera, Rose GF Leke, Diane W Taylor

**Affiliations:** 1 John A Burns School of Medicine, University of Hawaii at Manoa, 651 Ilalo St, Honolulu, HI 96813, USA; 2 Department of Microbiology, Faculty of Medicine, University of Panama, Apartado 0819-05523, El Dorado, Panama City, Panama; 3 Instituto de Inmunología, Edificio de Microbiología, Tercer Piso, Facultad de Salud, Universidad del Valle, Sede San Fernando, Cali, Colombia; 4 Faculty of Medicine & Biomedical Research, University of Yaoundé 1, Yaoundé, Cameroon

## Abstract

**Background:**

*Plasmodium falciparum *merozoite surface protein-1 (MSP1) has been extensively studied as a blood-stage malaria vaccine candidate, with most work focused on the conserved 19 kDa and semi-conserved 42 kDa C-terminal regions (blocks 16-17) and the hypervariable N-terminal repeat region (block 2). However, recent genotyping studies suggest that additional regions of MSP1 may be under selective pressure, including a locus of intragenic recombination designated as block 4 within the 3' region of the gene.

**Methods:**

The current study examined the antibody response to the two parental and two recombinant forms of block 4 and to blocks 16-17 (3D7) in study populations from Colombia, Papua New Guinea and Cameroon that differ in malaria transmission intensity and ethnic composition.

**Results:**

IgM and IgG antibodies were detected against parental and recombinant MSP1 block 4 peptides in all three populations. Overall, 32-44% of the individuals produced IgM to one or more of the peptides, with most individuals having IgM antibodies reactive with both parental and recombinant forms. In contrast, IgG seropositivity to block 4 varied among populations (range 15-65%), with the majority of antibodies showing specificity for one or a pair of block 4 peptides. The IgG response to block 4 was significantly lower than that to blocks 16-17, indicating block 4 is subdominant. Antibodies to block 4 and blocks 16-17 displayed distinct IgG subclass biases, with block 4 responses biased toward IgG3 and blocks 16-17 toward IgG1. These patterns of responsiveness were consistently observed in the three study populations.

**Conclusions:**

Production of antibodies specific for each parental and recombinant MSP1 block 4 allele in different populations exposed to *P. falciparum *is consistent with balancing selection of the MSP1 block 4 region by the immune response of individuals in areas of both low and high malaria transmission. MSP1 block 4 determinants may be important in isolate-specific immunity to *P. falciparum*.

## Background

*Plasmodium falciparum *merozoite surface protein 1 (MSP1) is a candidate antigen for inclusion in a blood stage malaria vaccine because it is thought to play a role in erythrocyte invasion [1]. The MSP1 gene has been divided into 17 sequence blocks that are conserved, semi-conserved, or variable [2]. The semi-conserved and variable regions are generally dimorphic, with the prototype MSP1 alleles represented by the K1 and MAD20 parasite isolates. Exceptions to this dimorphism are tripeptide repeat sequences comprising block 2, which are of variable length and composition, and a RO33 non-repetitive block 2 variant. In addition, the MSP1 gene contains several loci of intragenic recombination which represent another potential source of antigenic polymorphism [2]. While it has been suggested that intragenic recombination can occur throughout the MSP1 gene, recombination sites in the 5' region (conserved blocks 3 and 5, and variable block 4) and in the 3' region (block 17) have been identified [3]. Sequence analysis of 34 full-length MSP1 sequences has provided no evidence of recombination in blocks 6 through 16 [4].

In a previous study, the genetic composition of polymorphic MSP1 regions of *P. falciparum *obtained from Buenaventura, Colombia, an area of low, seasonal malaria transmission was examined [5]. There was restricted genetic diversity of MSP1 in this population, with a high level of conservation within blocks 2, 6, and 16-17 that corresponded exclusively to the MAD20 allelic type. In contrast, four MSP1 block 4 types corresponding to the K1 and MAD20 parental and recombinant sequences were detected. The persistence of both parental and recombinant alleles of block 4 despite the restricted heterogeneity throughout the rest of the gene suggested that block 4 allelic diversity may be under balancing selection.

This study examined the recognition of MSP1 block 4 by antibodies of humans exposed to *P. falciparum *infection in order to begin to address the potential immunological significance of MSP1 block 4 sequence variability. The study populations examined were from three different countries with varying *P. falciparum *transmission intensities. IgM and IgG antibodies to block 4 peptides were measured to determine the seroprevalence and magnitude of the block 4 responses. The specificity of antibodies produced against block 4 epitopes were characterized as cross-reactive or allele-specific, and thus capable of discriminating among parental and recombinant block 4 sequences. Finally, the IgG isotype distribution of antibodies specific for block 4 as compared to those specific for blocks 16-17 was compared. A basic question addressed in these experiments was: are antibodies specific for each parental and recombinant MSP1 block 4 allele found in different populations exposed to *P. falciparum*? If so, this would be consistent with balancing selection of the MSP1 block 4 region by the immune response of individuals in areas of both low and high malaria transmission.

## Methods

### Study populations

The patient samples examined were archived sera collected from regions of varying endemicity for *P. falciparum *malaria. All samples were derived from research protocols that had been approved by the appropriate Human Use Review Committees in the originating country as well as at the US collaborating institutions. Informed consent was obtained from each study subject prior to enrollment. Use of these samples for this study was approved by the University of Hawaii Committee on Human Studies.

#### Colombia (malaria uninfected and infected, asymptomatic)

The study site in Colombia was the village of Punta Soldado in the Department of Valle del Cauca. Malaria transmission in this area is considered to be low and seasonal. Serum samples were collected in 1993 from a study population consisting of 151 asymptomatic children and adults within the following age groups: 1-4 y (n = 10), 5-14 (n = 51), 15-29 (n = 44), and 30+ (n = 46)[6]. Fifty individuals (33.1%) were positive for *P. falciparum *by Giemsa-stained thick blood smears but were asymptomatic. Sufficient serum for analysis in this study was available for 111 of these samples.

#### Papua New Guinea (malaria uninfected and infected, asymptomatic)

The study site was located in a rural area of intermediate malaria transmission 13 kilometers south of Madang, Papua New Guinea. Sera were obtained in 1985-1986 from asymptomatic adult females at delivery (n = 101) and from cord blood (n = 71)[7]. The prevalence of malaria infection, predominantly *P. falciparum*, in the study population was 26% in mothers (mostly low density *P. falciparum*) prior to or immediately after delivery and 4% in cord blood samples. Of these samples, 22 adult and 9 cord blood sera were available for this study. Within this study population, 43.6% women reported a history of malaria symptoms sometime during their pregnancy [8].

#### Cameroon (malaria uninfected and infected, asymptomatic)

The study site was located in Ngali II, a rural Cameroonian village. Plasma was obtained in 2001-2004 from 72 healthy adult volunteers (mean age 36.1 y, age range 14-79 y), 37.5% of whom were blood smear positive for *Plasmodium falciparum. Plasmodium falciparum *transmission in this area is perennial and hyperendemic, with an estimated exposure rate of ~300 infectious bites per year. All 72 samples were tested for IgM antibodies, while 64 of these samples were tested for IgG antibodies due to limited availability of antigen-coated microspheres.

### Synthetic peptide and recombinant antigens

The malaria antigens used in the study included the recombinant MSP-1_42 _(3D7) polypeptide corresponding to MSP1 blocks 16-17 expressed in *Escherichia coli *(molecular weight, 42,000), produced by the Malaria Vaccine Development Branch (MVDB), National Institute of Allergy and Infectious Disease, National Institutes of Health, Rockville, MD, and a set of synthetic peptides corresponding to parental and recombinant forms of MSP1 block 4. Synthetic peptides were synthesized by Alpha Diagnostic International, Inc. (San Antonio, TX). Peptides corresponding to the two parental allelic forms of MSP1 block 4 were designated block 4-1 (K1 allele) and block 4-2 (MAD20 allele). Peptides corresponding to the two recombinant forms were designated block 4-3 (K1:MAD20) and block 4-4 (MAD20:K1). The sequences of the peptides were:

Block 4-1 - NENIKELLDKINEIKNPPPANSGNTPNTLLDKNKKIE (37-mer);

Block 4-2 - NENIKKLLEDIDKIKTDAENPTTGSKPNPLPENKKKEVEG (40-mer);

Block 4-3 - NENIKKLLDKINEIKNPPPANSGNTPNPLPENKKKEVEG (39-mer);

Block 4-4 - NENIKKLLEDIDKIKTDAEKPTTGSKPNTLLDKNKKIE (38-mer)

Antigens were coupled to SeroMAP™ microspheres according to a protocol previously described [9] using 5 μg of MSP-1_42 _or 20 ug peptide coupled to five million microspheres.

### Multiplex assay for antibodies

A Luminex^® ^bead-based multiplex serological assay was used to measure antibodies to the four block 4 peptides and to blocks 16-17 [9]. Briefly, antigen-coated microspheres were mixed with diluted plasma (1:100 dilution) in microtiter plates and incubated on a microplate shaker (Lab-line, Melrose Park, IL) for 1 hour at 500 rpm. After incubation, the plates were washed and microspheres resuspended in buffer containing 1 mg/ml R-phycoerythrin-conjugated, F(ab')2 fragment, goat anti-human IgG (Fcγ fragment specific) (Jackson ImmunoResearch, West Grove, PA) or R-phycoerythrin-conjugated, F(ab')2 fragment, donkey anti-human IgM (Fc5μ fragment specific) (Jackson ImmunoResearch, West Grove, PA). Plates were incubated on a microplate shaker for 1 hour, washed and resuspended. Resuspended microspheres were analysed using a Liquichip M100 reader (QIAGEN, Valencia, CA). For IgG subclass-specific assays, diluted plasma was incubated with antigen-coated microspheres in microtiter plates on a microplate shaker (Lab-line, Melrose Park, IL) for 1 hour at 500 rpm. After incubation, the plates were washed and microspheres resuspended in buffer containing the appropriate secondary antibody diluted to 1:1000 (mouse anti-human IgG1 Fc fragment specific (Calbiochem) or mouse anti-human IgG3 (Southern Biotechnology)). Plates were incubated on a microplate shaker for 1 hour, washed and resuspended in 100 μl of 1 μg/ml of R-phycoerythrin-conjugated, F(ab')_2 _fragment, donkey anti-mouse IgG (H+L) (JacksonImmunoResearch, West Grove, PA). In order to establish a cut-off for positive samples, frequency distribution curves for median fluorescent intensity (MFI) values were plotted for each antigen and the corresponding limits for negative and positive samples were determined. This allowed for a better assessment of the cut-off than the mean MFI plus three standard deviations of the negative serum controls. The following cut-off values were used in this study: block 4 peptides IgM: 800 MFI; block 4 peptides IgG: 600 MFI; blocks 16-17 polypeptide IgM: 500 MFI; blocks 16-17 polypeptide IgG: 2000 MFI; block 4 peptides and blocks 16-17 polypeptide IgG subclasses: 100 MFI.

### Statistical analysis

Mean MFI values were calculated for positive samples for each peptide or polypeptide antigen. The one-way ANOVA was used to compare IgM and IgG MFI values between and within populations. Comparisons between MFI values for individual peptide pairs were performed using the post hoc Tukey-Kramer honestly significant difference (HSD) test. Correlation of MFI values for pairs of peptides were calculated using Pearson's product-moment correlation coefficient (r). The significance of differences between the means of two data sets was calculated using Student's t-test. A *P *value of less than or equal to 0.05 was considered to be significant. All calculations were performed using the JMP software package (version 7.0.2, SAS Institute, Cary, NC).

## Results

### IgM response to parental and recombinant block 4 and blocks 16-17 peptides

**Figure 1:**
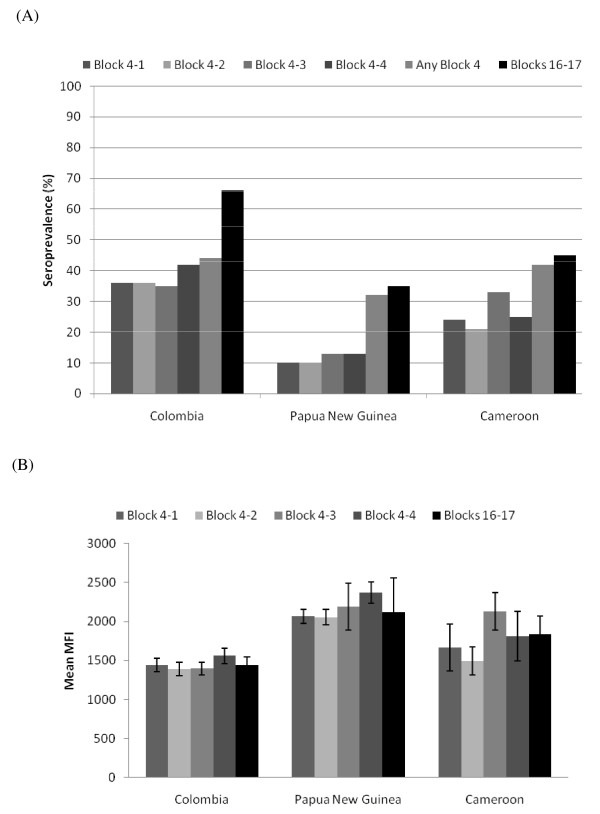
**IgM responses to MSP1 Block 4 and Blocks 16-17**. (A) Seroprevalence based on n = 111 Colombian, n = 22 Papua New Guinean and n = 72 Cameroonian samples. (B) Mean antibody levels (MFI units) for positive samples. Error bars correspond to the standard error of the mean.

Overall, seroprevalences ranged from 10 to 42% for individual peptides (Figure 1A), with 32% to 44% of individuals within each of the three populations having IgM antibodies to one or more block 4 peptide (Figure 1A). For comparison, the seroprevalence of IgM antibodies to blocks 16-17 ranged from 35% to 65% (Figure 1A). Within each population, the prevalence of IgM antibodies to each of the four block 4 peptides was similar, i.e., ~35% in Colombians, 10% in female adults in PNG, and 25% in Cameroonians. Additionally, when individuals had IgM antibodies against one peptide, their sera usually reacted with the other three peptides as well, suggesting cross-reactive epitopes shared among the peptides.

Antibody levels for positive samples within each population were also similar for all of the block 4 peptides (Figure 1B). In addition, IgM levels against the four block 4 peptides were similar among the populations. The only exception was block 4-3 (one-way ANOVA, F = 7.56, P = 0.0001), where block 4-3 IgM levels in Cameroonians were higher than in Colombians (Tukey-Kramer HSD, P ≤ 0.05).

Since the data suggest IgM block 4 antibodies were cross-reactive, the Pearson's correlation coefficient (r) was calculated for IgM antibody levels for each peptide pair (Table 1). There was a highly significant correlation between IgM MFI values for all pair-wise block 4 peptide comparisons (r = 0.3180-0.9963, *P *= .0065 to < 0.0001), consistent with a high level of cross-reactivity among IgM block 4 antibodies. This correlation was most striking in the case of the Colombian samples, for which a nearly 1:1 relationship (r = 0.96-0.98, P < 0.0001) existed for all block 4 peptide pairs.

**Table 1:** Pair-wise correlation of IgM and IgG MFI values for block 4 peptides and blocks 16-17*

Antigen Pair		Colombia		Papua New Guinea		Cameroon	
**IgM**		**r**	***P***	**r**	***P***	**r**	***P***

4-1	4-2	**0.966**	< .0001	**0.9888**	< .0001	**0.7644**	< .0001
4-1	4-3	**0.9663**	< .0001	**0.7354**	< .0001	**0.3792**	0.001
4-1	4-4	**0.9876**	< .0001	**0.9963**	< .0001	**0.6942**	< .0001
4-2	4-3	**0.9877**	< .0001	**0.8133**	< .0001	**0.9263**	< .0001
4-2	4-4	**0.9829**	< .0001	**0.9859**	< .0001	**0.659**	< .0001
4-3	4-4	**0.9764**	< .0001	**0.7318**	< .0001	**0.318**	0.0065

**IgG**		r	*P*	r	*P*	r	*P*

4-1	4-2	0.0888	0.3543	-0.1114	0.5507	0.1737	0.1699
4-1	4-3	**0.2389**	0.0116	**0.8043**	< .0001	**0.6795**	< .0001
4-1	4-4	0.0583	0.5432	-0.1443	0.4386	**0.3889**	0.0015
4-2	4-3	0.0253	0.7918	-0.036	0.8475	0.1993	0.1143
4-2	4-4	**0.7882**	< .0001	**0.8756**	< .0001	**0.2502**	0.0462
4-3	4-4	0.0031	0.9745	-0.105	0.5739	0.0976	0.443

*Pearson's product moment correlation coefficient (r) and corresponding *P *values. Significant r values are highlighted in bold print(*P *< 0.05).

### IgG responses to parental and recombinant block 4 and blocks 16-17 peptides

**Figure 2:**
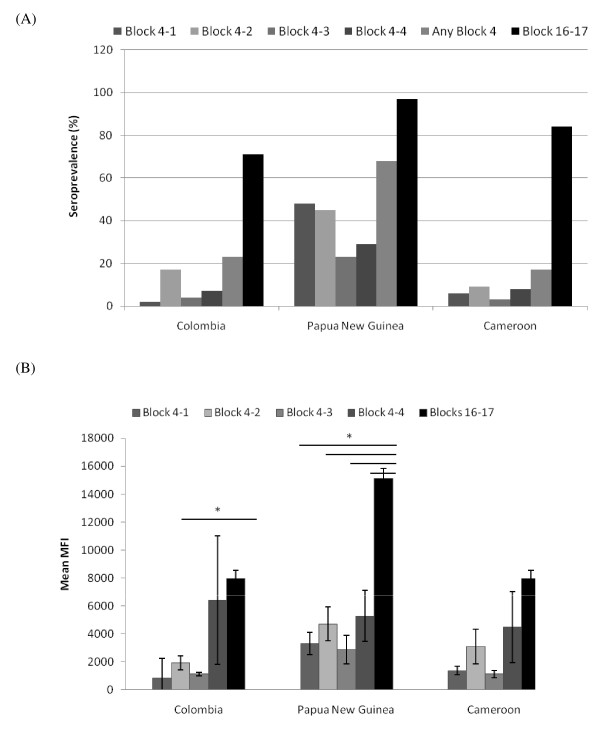
**IgG responses to MSP1 Block 4 and Blocks 16-17**. (A) Seroprevalence based on n = 111 Colombian, n = 22 Papua New Guinean and n = 64 Cameroonian samples. (B) Mean antibody levels (MFI units) for positive samples. Error bars correspond to standard error of the mean. Significantly different mean MFI values ( *P *≤ 0.05) are indicated with an asterisk.

The seroprevalences of IgG antibodies to block 4 peptides (Figure 2A) were lower than the IgM prevalences (Figure 1A) for all three populations, whereas the IgG seroprevalence was higher compared to IgM to blocks 16-17. These data suggest that IgG responses to block 4 epitopes are subdominant to those against blocks 16-17 in many endemic settings. In general, IgG in sera from PNG recognized the most alleles and had the highest seroprevalence compared to the other two sites. IgG responses to block 4-2 varied among the populations (one-way ANOVA, F = 4.16, P <0.0114), with IgG levels in PNG being significantly higher than in Colombia (Tukey-Kramer HSD, P ≤ 0.05). Only sera from individuals in PNG contained IgG antibodies that recognized all four block 4 peptides (Table 2). In contrast, 90% of sera from Colombians had IgG that was specific for only one block 4 peptide, particularly block 4-2, with only two samples recognizing multiple block 4 peptides (Table 2).

**Table 2:** Specificity of IgG antibodies for block 4 allelic forms in each study population and in all study populations^a^

Block 4 Peptide(s)	Colombia	Papua New Guinea	Cameroon	Total
1	9%	20%	9%	13%
2	68	15	27	40
3	9	0	0	6
4	4.5	0	27	7
1+2	4.5	5	0	4
1+3	0	5	0	2
1+4	0	5	9	4
2+3	0	0	0	0
2+4	4.5	10	9	7
3+4	0	0	0	0
1+2+3	0	10	9	6
1+2+4	0	10	9	6
1+3+4	0	0	0	0
2+3+4	0	5	0	2
1+2+3+4	0	15	0	6

^a^Percent positive based on IgG samples reacting with one or more block 4 peptides: 22/111 samples from Colombia, 19/22 from PNG, and 11/61 from Cameroon. Total: Percent positive based on a total of 52 responders from all groups

Both the seroprevalences and mean levels for IgG antibodies to individual block 4 peptides showed marked variation within each study population (Figure 2A &2B), thereby demonstrating that IgG anti-block 4 antibodies are allele-specific. The magnitude of IgG responses to block 4 tended to be lower than IgG responses to blocks 16-17, and these differences were statistically significant for all block 4 peptides for Papua New Guinea and for block 4-2 for Colombia (Figure 2B). Among block 4-specific antibodies, the predominant response was to the parental alleles, with the 68% of positive Colombian sera reacting with block 4-2 (MAD20); 20% and 15% of positive sera from PNG reacting with peptides 4-1 (K1) and 4-2 (MAD20), respectively. In contrast , 27% of Cameroonian sera reacted with peptides 4-1 (K1) and 4-4 (MAD20/K1 recombinant) (Table 2). A portion of the samples, however, contained antibodies that recognized both a parental and one recombinant block 4 peptide, usually blocks 4-1 + 4-3 and blocks 4-2 + 4-4 combinations Figure 2B, Table 2). No sample recognized the combination of blocks 4-2 + 4-3 or block 4-3 + 4-4 combination. These observations, along with the correlations observed in Table 1, suggest that in the IgG response (1) there are major block 4 epitopes that are unique to the two parental block 4 sequences, (2) there are epitopes that are shared between parent and recombinant forms (eg. between blocks 4-1 and 4-3 and between blocks 4-2 and 4-4, and (3) cross-reactive IgG antibodies are rare between the two parental and between the two recombinant block 4 peptides.

### Comparison of overall seroprevalence of block 4 and blocks 16-17 antibodies

**Figure 3:**
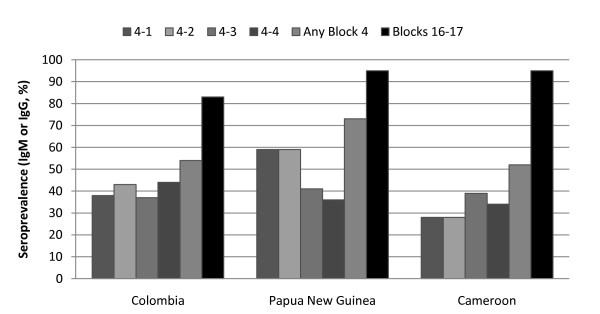
**Overall seroprevalence of IgM or IgG antibodies to MSP1 Block 4 and Blocks 16-17**. Seroprevalence based on n = 111 Colombian, n = 22 Papua New Guinea and n = 64 Cameroonian samples.

In considering the seropositivity of the various study populations, it is helpful to examine the total seropositivity (IgM + IgG) presented in Figure 3 since the responses were largely IgM responses in the low endemicity area (Colombia) but had switched to IgG responses in areas of higher endemicity (PNG and Cameroon). When IgM and IgG results were combined to obtain the overall seroprevalence of antibodies to the two MSP1 regions, the seroprevalence to individual Block 4 peptides ranged from 28 to 59% while seroprevalence to any Block 4 peptide ranged from 52 - 73%. This contrasted to an overall seroprevalance of 83 to 95% for Blocks 16-17 (Figure 3).

### IgG subclass distribution of block 4 and blocks 16-17 antibodies

**Figure 4:**
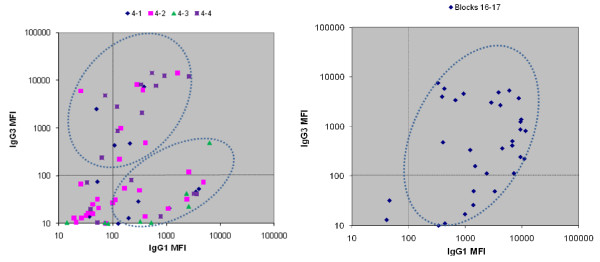
**IgG subclass distribution of MSP1 Block 4 and Block 16-17**. Sera from samples that contained antibodies to one or more Block 4 peptides (left panel) or Blocks 16-17 (right panel)  were assayed for IgG1 and IgG3 isotypes. Colombia (n = 21), Papua New Guinea (n = 9), and Cameroon (n = 11). Clustering of isotype distribution is indicated by ellipses.

Preliminary screening indicated that antibodies produced against both MSP1 regions were primarily of the IgG1 and IgG3 subclasses, consequently a subset of samples from each region was tested for these two isotypes (Figure 4). In this isotype-specific assay, IgG1 and IgG3 to block 4 were detected in 54% and 32% of the samples, respectively, with both isotypes being detected in 27% of the samples, IgG1 only in 33% , IgG3 only in 6.7%. IgG3 levels (mean MFI = 4,912), however, were significantly higher than levels of IgG1 (mean MFI = 1035, *P *= 0.0009 by Student's t test). The IgG isotype response to block 4 appeared to form two clusters, one cluster of samples with high IgG3 and low/no IgG1 and a second cluster with high IgG1 and low/no IgG3 (Figure 4). In contrast, IgG1 and IgG3 to blocks 16-17 were found in 89% and 59% of the samples, respectively, with both isotypes detected in 75.8% of the samples, IgG1 only in 18.1%, and none of the samples containing only IgG3. These samples appeared broadly dispersed in a single cluster. There were no differences between the mean MFI values for the two isotypes (IgG1 mean MFI = 3,587; IgG3 mean MFI = 2,169; *P *= 0.103) in the response to the MSP1 C-terminus. Furthermore, the mean IgG3 response to block 4 peptides (average MFI = 2,209) was significantly higher than the mean IgG3 response to blocks 16-17 (average MFI = 1,050) (*P *= 0.015, Student's t-test).

**Figure 5:**
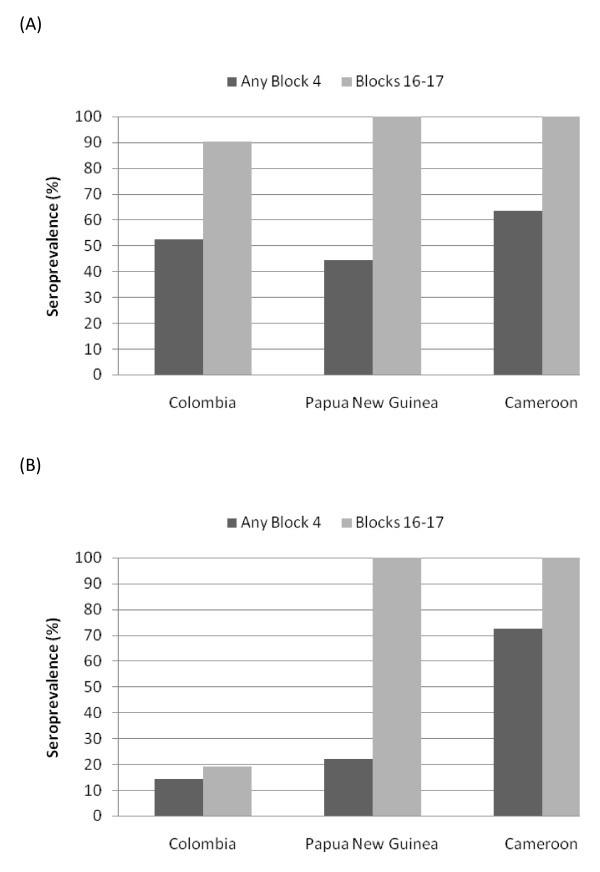
**IgG subclass responses to MSP1 Block 4 and Blocks 16-17**. (A) IgG1 and (B) IgG3 seroprevalences based on n = 21 Colombian, n = 9 Papua New Guinean and n = 11 Cameroonian samples.

IgG subclass data were also analysed by geographic region (Figure 5). When the IgG1 responses to block 4 and blocks 16-17 were compared for the three regions (Figure 5A), there was no significant pattern of variation in relation to transmission intensity. However, a similar comparison for the IgG3 responses revealed an increase in IgG3 seroprevalence with increasing transmission intensity, particularly for block 4 antibodies. Interestingly in Cameroon, the area of highest transmission intensity, the seroprevalence of block 4 IgG3 antibodies (73%) was higher that that of block 4 IgG1 antibodies (64%) in samples tested for IgG subclass.

### IgM response to parental and recombinant block 4 and blocks 16-17 peptides in cord blood

**Figure 6:**
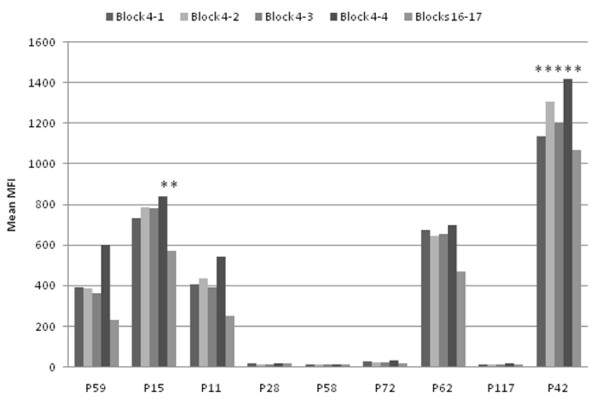
**Cord Blood IgM responses to MSP1 Block 4 and Blocks 16-17**. Mean antibody levels (MFI units) for cord blood samples from Papua New Guinea. Asterisks indicate samples that were above adult cut-off values for the individual peptides.

Nine cord blood samples were available from Papua New Guinea for evaluation of the response of the fetus to MSP1 antigens in utero. Two of these samples contained IgM antibodies that exceed adult background cutoff levels for one or more block 4 peptides and blocks 16-17 (P15 and P42) (Figure 6). However, five of the nine cord blood sera (P59, P15, P11, P62, P42) had measureable MSP1-specific IgM antibodies as compared to four sera (P28, P58, P72, P117) with undetectable levels of these antibodies, suggesting that fetal responses to both block 4 and blocks 16-17 are common following in utero exposure to *P. falciparum*.

## Discussion and Conclusions

Block 4 is one of the most clearly defined sites of intragenic recombination within the MSP1 sequence [4,10]. While extensive studies have analysed the immune responses to the MSP1 block 2 repeat region and blocks 16-17, few immunological studies have been performed on MSP1 block 4. Immune recognition of this region was first shown using a mouse monoclonal antibody that bound an isolate-specific epitope within block 4 [1,11]. Later, human antibodies were detected to one allelic form of block 4 in sera of German adults following *P. falciparum *infection and in Burkina Fasoan infants and adults [12]. The seroprevalence of anti-block 4 IgG antibodies was lower in German adults undergoing a primary (14%) or subsequent (21%) responses compared to Burkina Fasoan infants (50%) following initial malaria infections. Subsequently, allele-specific antibodies to MSP1 block 4 were detected in sera of malaria-exposed individuals in a study utilizing a panel of MSP1 recombinant polypeptides that included peptides spanning block 4 of the K1, MAD20 and RO33 parasites isolates [13]. IgG reactive with MAD20, K1 and RO33 block 4 were detected in children (1-5 y), adolescents (6-14 y), and adults (>14 y). The seroprevalence of antibodies was similar for all three block 4 sequences in adults, while the responses were more variable among children and adolescents. However, little was known about the specificity and cross-reactivity of IgM and IgG block 4 antibodies prior to the current study.

In this study, immune recognition of dimorphic and recombinant MSP1 block 4 peptides by IgM and IgG antibodies from malaria-exposed subjects from Colombia, Papua New Guinea and Cameroon was evaluated. For comparison, antibody responses to the MSP1 C-terminal 42 kDa region (blocks 16-17), which is highly immunogenic in malaria-infected individuals [14] were examined. Despite the diverse characteristics of the study populations, a consistent pattern emerged with respect to IgM and IgG antibody recognition of block 4 and blocks 16-17. Sera from all three populations contained IgM and IgG antibodies to the block 4 synthetic peptides and to blocks 16-17. For IgM, MSP1 block 4 and blocks 16-17 seroprevalences in most instances were similar ranging from 44% and 66% in Colombia, 32% and 35% in Papua New Guinea, and 40% and 43% in Cameroon, respectively. In contrast, IgG seroprevalences were much lower for MSP1 block 4 than for blocks 16-17 in the three populations. When IgM and IgG recognition are combined, more than half of the individuals from each population produced antibodies recognizing MSP1 block 4, while more than 80% produced antibodies to MSP1 block 16-17.

The similarity in prevalence and magnitude of the IgM response to MSP1 block 4 and blocks 16-17 suggests that these regions are equi-dominant during the initial immune response in contrast to dominance of a later MSP1 IgG response specific for the blocks 16-17. Thus, it appears that exposure to *P. falciparum *MSP1 through natural infection in all endemic settings generally resulted in the preferential expansion of IgG-producing B cells specific for blocks 16-17 over those specific for MSP1 block 4. Studies on humans naturally exposed to malaria infection have reported high seroprevalence levels of antibodies against MSP1 C-terminal epitopes [12,14-16].

The IgM response, which reflects the repertoire of early B cells that have not undergone isotype-switching, was highly cross-reactive among the parental and recombinant block 4 sequences as demonstrated by pair-wise comparisons (Table 1). This high level of cross-reactivity is remarkable since only ~28% sequence homology exists among block 4 parental and recombinant sequences. IgM antibodies may be directed against amino acid residues shared by all four block 4 sequences or, alternatively, may display low affinity binding of similar conformational determinants preserved among the alleles by conservative amino acid substitutions. It is notable that cross-reactive block 4 IgM antibodies were also detected in cord blood samples from Papua New Guinea indicating that these antibodies may be produced by the foetus during the response to malaria antigens in utero.

**Figure 7:**
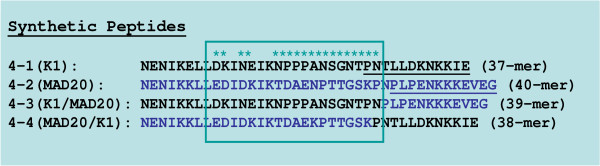
**Mapping of dimorphic allelic IgG MSP1 Block 4 epitopes**. Human antibodies that reacted with Block 4-1 and 4-3 (black) and with Block 4-2 and 4-4 (blue) recognized allelic sequences indicated by asterisks within the boxed region.

The IgG block 4 response was specific and clearly discriminated among MSP1 block 4 sequences. A majority of individuals produced IgG antibodies specific for one of the two parental block 4 peptides (i.e., K1 or MAD20) and these antibodies may contribute to isolate-specific immunity to *P. falciparum*. A subgroup of samples had IgG antibodies that cross-reacted with epitopes shared by one parental and one recombinant allele. Pair-wise comparisons of IgG levels to MSP1 block 4 peptides (Table 1), as well as the specificity of individual samples (Table 2) mapped these two allelic epitopes to dimorphic sequences localized within a 19 amino acid region upstream of the putative block 4 recombination site (Figure 7). However, in all three study populations, IgG antibodies highly specific for the prototype K1 (Block 4-1) and MAD20 (block 4-2) sequences were more prevalent than antibodies recognizing the recombinant (block 4-3 and 4-4) forms.

The Colombian samples examined in this study were obtained from subjects residing in relatively close proximity to the site where *P. falciparum *MSP1 genotyping had been performed [5]. Parasites expressing all four MSP1 block 4 sequences were found in this low-transmission region. In the current study, Colombian sera exhibited the highest block 4 IgM seroprevalence (Figure 1A), the highest cross-reactivity of IgM for the four peptide pairs (Table 1), but the most restricted IgG specificity for single block 4 peptides compared to the other two higher-transmission regions. In Papua New Guinea and Cameroon, where higher malaria transmission occurs, a much broader range of specificities developed, including a higher proportion of sera containing IgG that recognized two or more block 4 allelic sequences.

IgG subclass analysis revealed that MSP1 block 4 and blocks 16-17 epitopes also differ in isotype switching preference. In general, the majority of samples contained both IgG1 and IgG3 antibodies to blocks 16-17, whereas, the response to block 4 was more polarized, with one cluster of samples containing either high levels of IgG3 with or without lower levels of IgG1 and a second cluster of samples containing predominantly IgG1. While block 4 induced both IgG1 and IgG3 antibodies, a greater proportion of these antibodies are biased toward a high IgG3 response than observed for blocks 16-17. When IgG subclass data were analysed by geographic region, it appeared that production of IgG3 antibodies, particularly against block 4, was dependent on malaria transmission intensity. A relatively high IgG1 and low IgG3 seroprevalence was seen against block 4 in the area of lowest malaria transmission while the prevalence of IgG3 antibodies was higher in the intermediate transmission region and maximal in the area of highest transmission, exceeding the IgG1 seroprevalence.

The potential relevance of the MSP1 block 4-specific antibody response to protective immunity remains to be explored. Antibodies against the MSP1 83 kDa processing fragment containing block 4 can inhibit the growth of *P. falciparum *[17], as well as block the activity of inhibitory antibodies to the MSP1 C-terminus [18]. MSP1 block 2 antibodies, which are predominantly IgG3[19], are highly efficient in mediating the monocyte-dependent antibody-mediated cellular inhibition (ADCI) of *P. falciparum *[20]. It has been suggested that variant MSP1 epitopes, such as the block 2 repeat region, are more likely to stimulate B cells that class switch to IgG3 production, and that these antibodies may be more effective in mediating ADCI [19,20]. Since many serum samples recognizing MSP1 block 4 peptides contained high levels of IgG3 in the current study, these block 4 antibodies may exert biological activity against blood stage parasites through monocyte-mediated inhibition of parasite growth.

The observation that individuals in the low transmission region produce antibodies against only a single block 4 peptide while those from intermediate and high malaria transmission regions produce antibodies against multiple block 4 peptides suggests that antibodies against the complete array of block 4 sequences may be associated with increased immunity to malaria. Differences in seropositivity among small numbers of individuals primed by natural infection may allow parasites expressing certain block 4 alleles to survive in individuals lacking antibodies against those particular alleles. Thus, balancing selection, ie. frequency dependent immune selection of block 4 peptide alleles within the parasite population, would exist at all transmission intensities but may be more forceful in areas of higher malaria transmission. Given this scenario, a polyvalent block 4 vaccine should induce immunity to all four alleles, even in individuals naturally primed against only one or a few block 4 alleles.

Subdominant epitopes play an important role in immunity to viral pathogens [21-24] and it has been suggested that subdominant epitopes may be important in immunity to *Plasmodium *[25]. This possibility is particularly pertinent in view of the lack of protective efficacy of a highly-immunogenic MSP1 42 kDa C-terminal vaccine in children in a recent Phase IIb trial [26]. To date, the only blood-stage malaria vaccine that has been documented to provide partial protection in a Phase I-2b trial, is the Combination B vaccine containing MSP1 blocks 3 and 4 fused to a universal circumsporozoite protein T cell epitope along with merozoite surface protein-2 and the ring-infected erythrocyte surface antigen [27]. Genetic analyses indicating that MSP1 block 4 may be a locus of balancing selection along with the results of the current study demonstrating IgG discrimination among block 4 allelic peptides and increased production of anti-block 4 antibodies of certain IgG subclasses in areas of high malaria transmission suggest that the immune response to MSP1 block 4 epitopes is biologically and evolutionarily relevant and may contribute to the protective antibody response to *P. falciparum*.

## Competing interests

The authors declare that they have no competing interests.

## Authors' contributions

SPC conceived of and coordinated the study, performed the data analysis and drafted the manuscript. AKKK carried out the immunological assays. ZIT, SH and RGFL coordinated and participated in sample and data collection. DWT contributed to the study design and helped to draft the manuscript. All authors read and approved the final manuscript.
